# A Through-Hole Lead Connection Method for Thin-Film Thermocouples on Turbine Blades

**DOI:** 10.3390/s19051155

**Published:** 2019-03-07

**Authors:** Jinjun Deng, Weihua Wang, Liuan Hui, Jietong Zhang, Xinhang Jin

**Affiliations:** Key Laboratory of Micro/Nano Systems for Aerospace, Ministry of Education, Northwestern Polytechnical, University, Xi’an 710072, China; jswangweihua@mail.nwpu.edu.cn (W.W.); huiliuan@mail.nwpu.edu.cn (L.H.); zhangjiet@mail.nwpu.edu.cn (J.Z.); jxh@mail.nwpu.edu.cn (X.J.)

**Keywords:** wire lead, thin film, thermocouple, through-hole lead connection

## Abstract

To solve the current problems with thin-film thermocouple signals on turbine blades in ultra-high temperature environments, this study explores the use of a through-hole lead connection technology for high-temperature resistant nickel alloys. The technique includes through-hole processing, insulation layer preparation, and filling and fixing of a high-temperature resistant conductive paste. The through-hole lead connection preparation process was optimized by investigating the influence of the inner diameter of the through-hole, solder volume, and temperature treatment on the contact strength and surface roughness of the thin-film for contact resistance. Finally, the technology was combined with a thin-film thermocouple to perform multiple thermal cycling experiments on the surface of the turbine blade at a temperature of 1000 °C. The results show that the through-hole lead connection technology can achieve a stable output of the thin-film thermocouple signal on the turbine blade.

## 1. Introduction

Along with the development of high power-to-weight ratio engine technology, the temperature in front of aero-engine turbine blades continues to increase, forcing engine components to function in a harsh environment for longer periods of time. The temperature in front of the turbine blade and turbine blade temperature are important parameters for evaluating the engine state, therefore, it is important to obtain the temperature parameters for the hot end of the runner components in aero-engines accurately and efficiently [1,2,3,4].

High-temperature instantaneous measurement of aerospace equipment requires a sensor which has both high temperature resistance and fast response. One micro-miniature sensor type is the thin-film thermocouple, which is made of materials with high temperature resistance and micro-electro- mechanical system (MEMS) technology, and is a promising test method [5,6,7]. Thin-film thermocouples are directly prepared on the surface of the structure using thin film deposition techniques, and include an insulating layer and device layer to achieve in situ measurements of the surface transient temperature. As the thickness of the thin-film thermocouple is on the order of micrometers, it has the advantages of small thermal inertia, fast response, and minimal interference with the test environment. Indium tin oxide (ITO) thin-film thermocouples are a ceramic-type thermocouple based on a wide band gap N-type semiconductor oxide thermoelectric material. The ITO thin-film thermocouple does not oxidize in high-temperature environments and has good stability at high temperatures. It is suitable for application as a thermocouple electrode material in harsh test environments with high temperature, high voltage, and transient temperature changes [8,9,10,11].

According to the working principle of the thin-film thermocouple, the lead wire is an important channel for transmission of the electrical signal. Accurately obtaining the electrical signal is necessary for subsequent analyses, and calculation of the voltage at the cold and hot ends can be used to determine the Seebeck coefficient of the thermocouple. The Seebeck coefficient is a working parameter of the thermocouple and is important for evaluating its performance. Regardless of the accuracy and response speed of the front-end thermocouple design, if the lead and film are in poor contact or fall off, the data collected will not accurately reflect the current temperature or thermocouple properties. At present, the main problems with wire bonding are that the solder can easily burn through the film, the wire bonding strength is low, and the wire exposed to high temperature environments can easily be damaged during gas flushing.

The Hewlett-Packard Company and NASA have used thermo-compression bonding to fix platinum-iridium thermocouple wires. This solution ensures the consistency of the materials at the lead connection, but the welding force is insufficient [12]. Techniques including thermal spray stacking, bolting machinery lead connections, and ultrasonic welding have also been explored [13]. However, none of these have provided a suitable wire connection method for the film sensor on turbine blades.

Holanda and others use parallel gap welding technology [14,15,16]. In this technique, the lead joint melts instantaneously under the thermal resistance of the mechanical pressure and pulse current, which connects the lead to the film; however, the resulting solder joint affects the flow field distribution and solder joint life. A high-temperature inorganic glue coating can effectively solve the problem of curved surface lead connections, and has the advantages of low cost, high efficiency, a firm connection, and good insulation effect. However, the electrical conductivity at the joint is poor, and the contact resistance is high. Therefore, the conventional lead method cannot reliably output a temperature measurement signal, and the advantages of the small size of the thin-film thermocouple device and its measurement of in situ deposition on the flow field without interference cannot be sufficiently exploited.

This study focuses on an analysis of the problems and current status of thin-film thermocouple leads and films. Considering anti-vibration properties, erosion resistance, and high lead connection strength, a through-hole lead connection technology is designed which largely solves the problems with using ITO and indium oxide thin-film thermocouple leads for temperature measurements of turbine blades.

## 2. Design and Fabrication of the Through-Hole Lead Connection

### 2.1. Design of the Through-Hole Lead Connection

The design of the through-hole lead connection structure is derived from the through-silicon via (TSV) three-dimensional package structure in MEMS technology. The TSV structure reduces the number of flying wires on the chip surface. It also reduces the chip size and power loss by filling the silicon via with conductive materials, which act as channels for electrical signals between the chip boards. This study draws on the concept of TSV three-dimensional packaging design to prepare the through-hole structure at the turbine blade and pass the lead through the through-hole to connect with the thermocouple film. The through-hole structure can provide protection for the sensor lead, in which the lead is protected by the cooling airflow. In areas of medium and low temperature, the lead avoids the gas routing of the turbine blade surface.

First, an insulating coating is prepared on the turbine blade surface, and thin film thermocouples are prepared on the surface of the insulating layer, extending the cold end (reference end) to the leading edge of the blade. Through-holes are machined on the leading edge of the blade. To ensure electrical insulation between the leads in the holes and the blades of nickel-based alloy, an alumina micro-ceramic tube is inserted into the holes and bonded using a high-temperature cement having good thermal matching properties.

The upper end of the lead must be reliably connected to the thin film thermocouple working under high temperature and variable cycle conditions to prevent the lead joint from creeping due to thermal expansion and contraction, resulting in separation from the film. In this study, high-temperature platinum paste is used to connect the lead joint and the cold end of the thermocouple film. Platinum paste (Pt: 99.9 wt.%) can increase the electrical contact area between the lead tab and the cold-end film, allowing the lead to output electrical signals more reliably. The platinum paste can also protect the nickel metal lead end after curing. This reduces the extent to which the lead connections in a gas environment are oxidized at high temperatures.

When routing on nickel-based alloy structural members, it is necessary to ensure that the leads can withstand higher temperatures (the actual operating temperature range of the turbine blades is 600–750 °C) and that the electrical insulation between the leads and the substrate can be maintained. To satisfy these requirements, an insulating coating with high temperature resistance should be prepared on the surface of the lead; this coating should have good thermal compatibility with the alloy lead and should not affect the toughness of the lead itself. The resistant insulating coating should not fall off when the lead is bent. In this study, a 10-μm-thick Al_2_O_3_ insulating case is fabricated on the leads to insulate the leads from the metal substrate when the wires are routed on the turbine.

**Figure 1:**
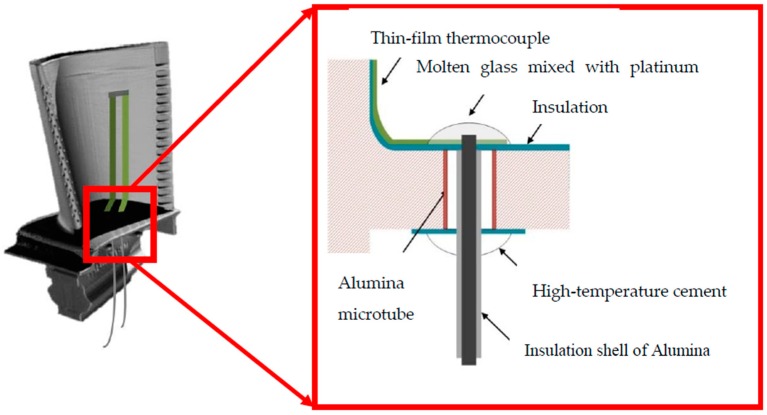
Structural diagram of the turbine blade through-hole lead connection.

High-temperature cement is an inorganic ceramic-based adhesive which is used for ultra-high temperature insulation and is suitable for bonding under environments of alternating high and low temperatures. Therefore, high-temperature cement is used to tightly fix the lead to the lower end of the separator. The through-hole lead connection structure is shown in Figure 1.

### 2.2. Fabrication of the Through-Hole Lead Connection

Based on the above analysis, a turbine blade is selected as the experimental subject, and the thin-film thermocouple is deposited on the turbine blade. The basic process includes surface cleaning of the blade substrate and preparation of the insulation layer preparation, followed by preparation of the sensor film and wire bonding on the blade. According to the design requirements discussed above, the following process steps can be designed:

#### 2.2.1. Surface Treatment and Processing

First, the substrate surface is treated. A small grinding wheel and shot blasting technology is used to remove oil stains and surface deposits. The resulting clean surface of the blade will ensure the effectiveness of the subsequent processes. The position of the through-hole is determined according to the position of the thermocouple electrode, and the through-hole is processed with an electric spark at the leading edge of the blade.

#### 2.2.2. Insulation Layer Preparation

Because the thin-film thermocouple is an electrical sensor device that converts a temperature difference signal into an electrical signal, it is necessary to insulate the substrate.

1. Insulating lead preparation

Nickel leads have the advantages of high electrical conductivity and high strength, and thus are used for lead connection to thermocouples. In this case, a nickel lead with a purity of 99.5% and diameter of 300 μm is selected. First, the substrate of a nickel wire is pre-processed. Then, the aluminum oxide coating is mixed with absolute ethanol, uniformly coated on the lead using a film-pulling machine, and dried at room temperature. Finally, sintering at 600 °C is performed to prepare the insulation covering on the surface of the lead.

2. Composite insulation layer preparation

**Figure 2:**
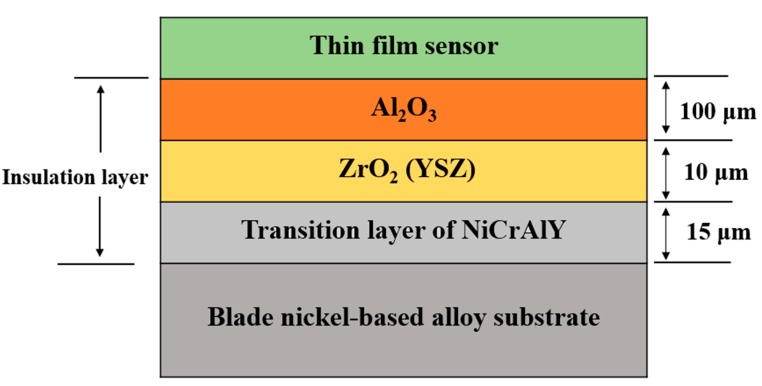
Structure of the composite insulating layer.

A composite insulating layer is designed, consisting of the turbine blade, a metal bonding layer, a thermal stress buffer layer, and an insulating layer. Of these, Al_2_O_3_ provides the primary insulation performance, and its thermal expansion coefficient differs from that of the superalloy substrate. Therefore, a ZrO_2_ oxide (YSZ) transition layer is prepared to buffer the thermal stress between the layers (the Al_2_O_3_ thermal expansion coefficient is 8 ppm/°C, the nickel-based superalloy thermal expansion coefficient is 13 ppm/°C, and the YSZ thermal expansion coefficient is 10 ppm/°C). In addition, to prevent destruction of the Ni-based superalloy substrate by oxidative diffusion, while at the same time improving the bonding force between the YSZ ceramic film layer and the metal substrate, a NiCrAlY alloy is used for the base. In summary, a composite insulating film layer is prepared by plasma spraying, including a 15-μm-thick NiCrAlY metal bond transition layer, a 10-μm-thick YSZ thermal stress buffer layer, and a 100-μm-thick Al_2_O_3_ insulating layer. The surface roughness of the insulating layer is 0.5 μm. The structure of the composite insulating layer is shown in Figure 2.

3. Insulation through-hole preparation

Because the inner diameter of the through-hole is small, the sprayed particles of the insulating layer cannot adhere to the inner wall of the through-hole. Thus, an alumina ceramic micro tube is fixed in the through-hole.

#### 2.2.3. Surface Graphics

**Figure 3:**
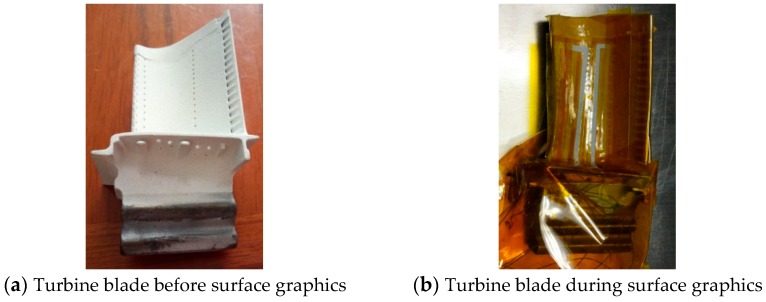
Surface graphic process.

A mask was prepared directly on the polyimide (PI) using an engraving apparatus. The PI film is attached to the surface of the workbench, and residual air bubbles are squeezed out to ensure uniform bonding. The graphic tool position file is imported to the engraving machine, the cutting contact force and tool path movement speed are set, the machining origin is specified, and the cutting is started. After the engraving is completed the PI film is removed and transferred to the surface of the blade. The turbine blade is then incubated at 80 °C for 0.5 h to improve the bonding force between the mask and the blade surface. The surface graphics is shown in Figure 3.

The patterned turbine blade is then placed in a magnetron sputtering apparatus, the argon gas flow rate and radio frequency voltage are set, and one side of the electrode is prepared. These steps are then repeated for the other side of the electrode.

#### 2.2.4. Lead Connection

**Figure 4:**
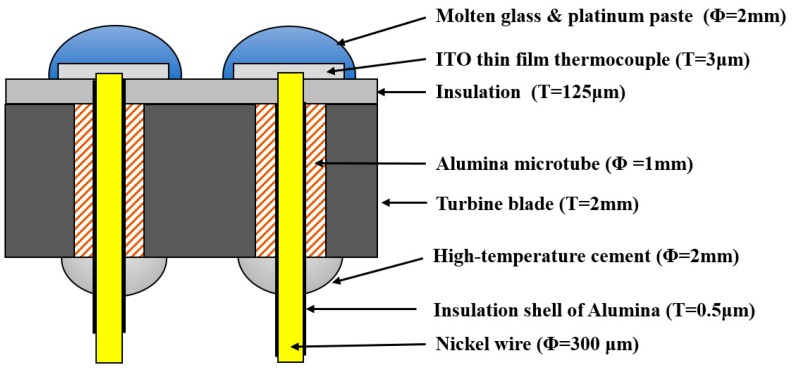
Lead connection diagram.

A nickel lead with an insulating layer is inserted into the ceramic tube, the upper end of the lead is polished to remove the insulating layer, and the lead terminal is fixed using molten glass mixed with platinum paste. As a result, the lead and the ITO thin-film thermocouple electrode are electrically connected through the solidified high-temperature platinum paste, while the connection between the lower end of the lead and the turbine blade is fixed with high-temperature cement, as shown in Figure 4. The structures’ dimension are shown in the Figure 4, where T is the thickness and Φ is the diameter of structure.

## 3. Lead Connection Feature Experiments

### 3.1. Lead Connection Strength Experiment

The Inconel 718 high-temperature nickel-based alloy is widely used in aerospace engines, and was selected as the experimental substrate in this study. The lead is close to the thermocouple, and the end of the thermocouple is fixed with molten glass. The roughness of the ITO thermocouple film is different, and the effect of its bonding with the molten glass is also different, and thus the surface roughness will affect the lead connection. The diameter of the through-hole is different, and the amount of high-temperature cement used for filling is thus also different. Therefore, to facilitate processing and comparison, the same through-holes are prepared, and alumina ceramic microtubes with the same outer diameter and varying inner diameters are fixed in the through-holes. The processing temperature of the high-temperature cement is also different, which affects the joint strength. Therefore, these three factors (the inner diameter of the ceramic tube, D, the surface roughness of the insulating layer, Ra, and the heat treatment temperature of the cement, T) are related to the strength of the lead connection, and orthogonal experiments are designed for these three factors.

The insulating layer is processed on the substrate to prepare a through-hole with a diameter of 1.2 mm, and ceramic tubes with an outer diameter of 1 mm and varying inner diameters are fixed in the hole. The alumina ceramic microtube is not fixed in the control group. The ITO film is processed using a magnetron sputtering machine and polished to varying roughness levels. The lead wire is inserted into the through-hole and bonded with high-temperature cement. After heat treatment at different temperatures, the tensile force is measured using a YZC-516C digital tensile test stand. The parameters are showed in Table 1 and test results are summarized in Table 2.

The visual analysis method for the orthogonal test is simple, intuitive, and computationally intensive. However, it cannot estimate the test error. In other words, it cannot distinguish whether the observed difference in the test results is caused by the change in each factor or by random fluctuation in the test. To solve this problem, an analysis of variance is performed on the test results. By calculating the squared sum of the factors and the significance level, a quantitative analysis of the influence of different experimental factors on the joint strength can be obtained, as summarized in Table 3. The F values in Table 3 represent the significance level, and it can be calculated by Equation (1). When the F value is greater than 1, it means that the factor has a significant effect on tensile strength, and it is marked as “*”. When the F value is less than 1, it means that the factor has no significant effect on tensile strength, and it is marked as “-”.

(1) $$F=\frac{SS_{j}}{SS_{error}}$$

The *SS_j_* means the sum of squares of deviations in column *j* in orthogonal test table, and it can be defined by the following Equation (2):

(2) $$SS_{j}=\frac{r}{n}\left(\overset{r}{\underset{i=1}{\sum }}K_{i}^{2}\right)-P$$

where *r* is the level of test factors (for this experiment *r* = 4), *n* is the total number of experiments (*n* = 16), and *P* is the mean of the sum of squares.

And the *SS_error_* is Equation (1) means the total dispersion square sum of experimental errors, and it can be defined by Equation (3):

(3) $$SS_{error}=\overset{16}{\underset{i=1}{\sum }}{\left(Fs_{i}-\bar{Fs}\right)}^{2}-\left(SS_{D}+SS_{Ra}+SS_{T}\right)$$

where *Fs_i_* is the tensile force of each test in Table 2, and $\bar{Fs}$ is the mean value of tensile force. *SS_D_*, *SS_Ra_* and *SS_T_* represent the sum of squares of deviations of diameter, surface roughness, and drying temperature, in Table 2, respectively.

**Figure 5:**
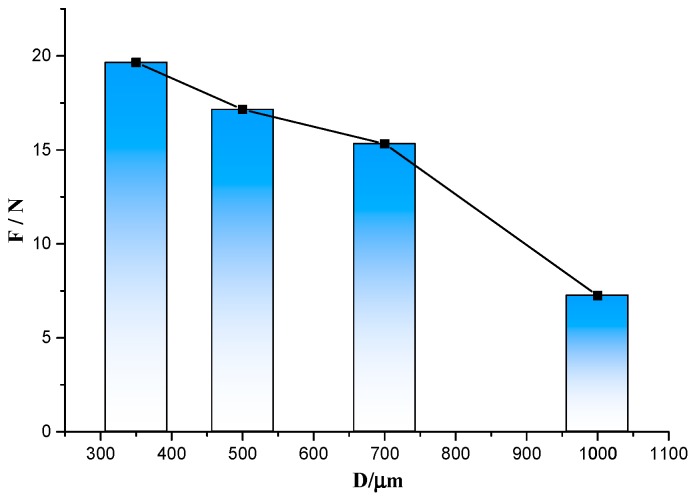
Relationship between the inner diameter and tensile force.

As presented in Table 3, the degree of influence of the three experimental factors on the lead connection strength is in the order of *D* > *Ra* > *T*, and the effect of the through-hole inner diameter is the most obvious. The tensile force for the same inner diameter is averaged, and the relationship between the inner diameter and the average tensile force is plotted, as shown in Figure 5.

Analyzing the relationship between the inner diameter and the strength through these experimental parameters, it can be seen that the joint strength increases with decreasing inner diameter.

### 3.2. Studies on the Influence of the Insulation Surface Roughness on Contact Resistance

The composite insulating layer prepared by plasma spraying has a large surface roughness, with a local average roughness Ra > 5 μm, and the thickness of the ITO thin-film thermocouple electrode layer is 2 μm. Therefore, it is possible to deposit ITO thin-film thermocouples on the insulating layer using a magnetron sputtering device. However, uneven deposition can result in different resistance values at the junction. These experiments explore the relationship between the roughness of the insulation layer and the ITO resistance.

**Figure 6:**
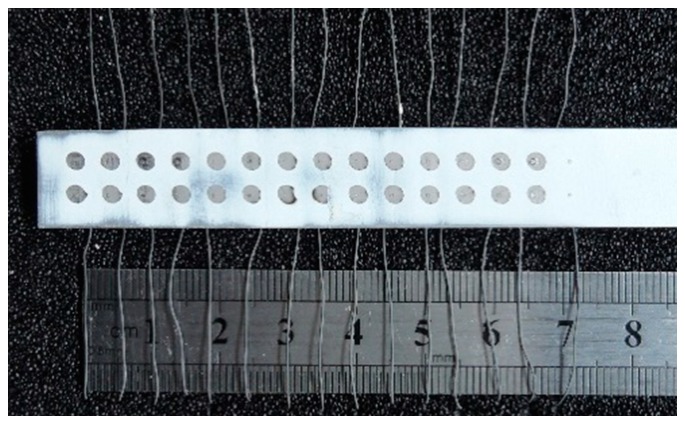
Photo of the through-hole lead connection.

Again, an Inconel 718 nickel-based superalloy plate is used as the experimental object. A total of 12 cylindrical through-holes with a diameter of 1.2 mm are machined on the substrate, and the insulating layer is processed. The surfaces of the insulating layers are polished with 1600#, 800#, 600#, or 240# sandpaper, and the roughness reading is consistent for every three measuring points, which are divided into four groups. High-temperature cement was used to fix the alumina ceramic tubes and leads, as shown in Figure 6. An ITO film with a diameter of 3 mm and thickness of 3 μm is prepared by magnetron sputtering, and a nickel wire is fixed on the edge of the ITO film using a conductive silver paste; the resistance between the nickel wire and the back wire is then measured. The resistance and roughness of each point are shown in the Table 4.

**Figure 7:**
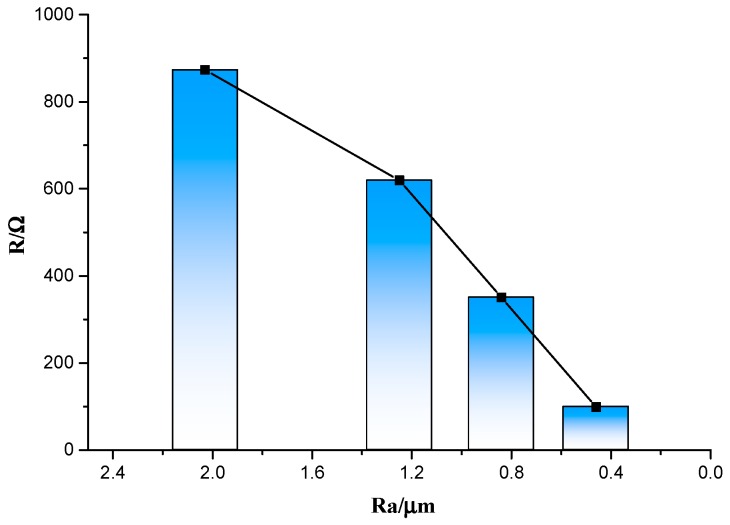
Relationship between roughness and lead connection average resistance.

Besides, the histogram of roughness and average resistance is plotted in Figure 7. It can be seen that the surface roughness of the insulating layer is positively correlated with the resistance of the through-hole lead connection lead, i.e., the smaller the surface roughness, the lower the uncertainty of the measured resistance will be. When the surface roughness is less than 0.5 μm, the through-hole lead connection structure has a resistance of <50 Ω, and the uncertainty is <±20 Ω. These values are much smaller than the ITO thin-film thermocouple resistance, which is on the order of thousands of ohms. Therefore, to reduce the resistance, the roughness should be reduced as much as possible during preparation of the insulating layer.

## 4. High Temperature Static Experiment

**Figure 8:**
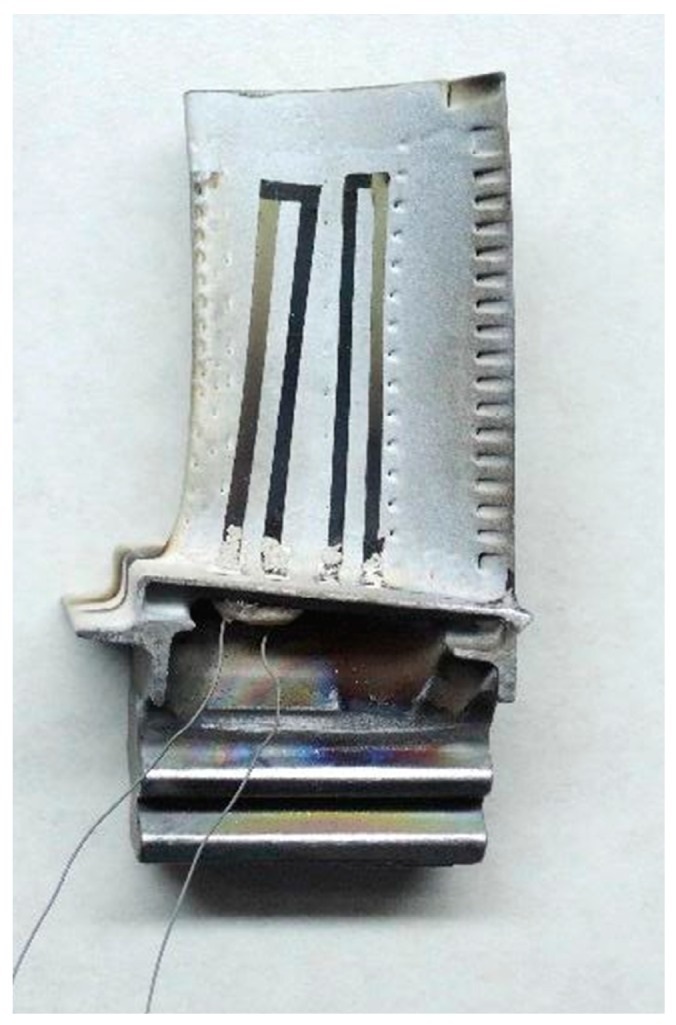
Photo of the turbine blade through-hole lead connection.

First, the surface of the turbine blade is cleaned to remove oil and dust, and the through-hole on the blade is processed by electric spark. Then, the composite insulating layer is processed, the roughness is polished, and the alumina ceramic microtube is fixed in the through-hole. The amine tape is patterned, a thermocouple is prepared in a vacuum magnetron sputtering apparatus, and an insulating layer is processed on the nickel lead. The lead wire is passed through the through-hole, the insulating layer is removed near one end of the thermocouple, the lead wire and the thermocouple are connected by molten glass, and the other end is fixed with high-temperature cement. The physical diagram is shown in Figure 8.

**Figure 9:**
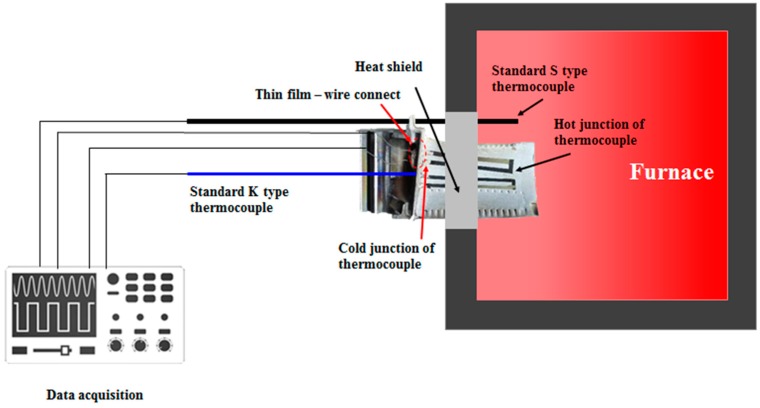
Schematic diagram of the experimental setup.

A K-type thermocouple is installed at the working and cold ends of the thermocouple, and the turbine blades are subjected to thermal cycling tests. The turbine blades are installed in a tubular vacuum furnace, the temperature is set to 1000 °C, and four temperature cycling events are carried out. After the temperature is raised, the elevated temperature is maintained for 1 h, after which the temperature is lowered to 700 °C. This cycle is repeated three times; during the last cycle, the temperature of 1000 °C is held for 3 h. The experimental setup is shown in Figure 9.

**Figure 10:**
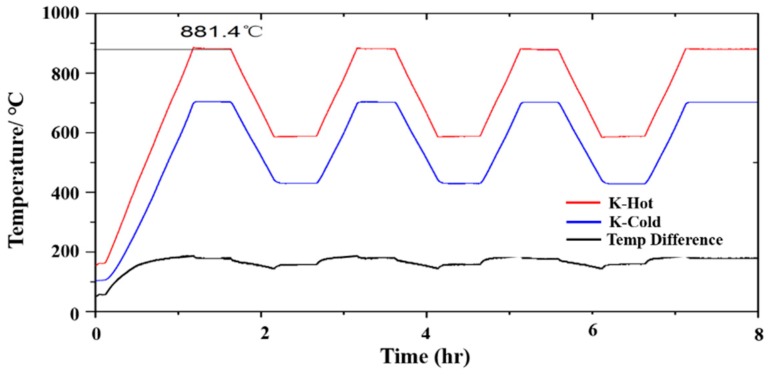
The hot junction temperature, clod junction temperature, and temperature difference during testing.

**Figure 11:**
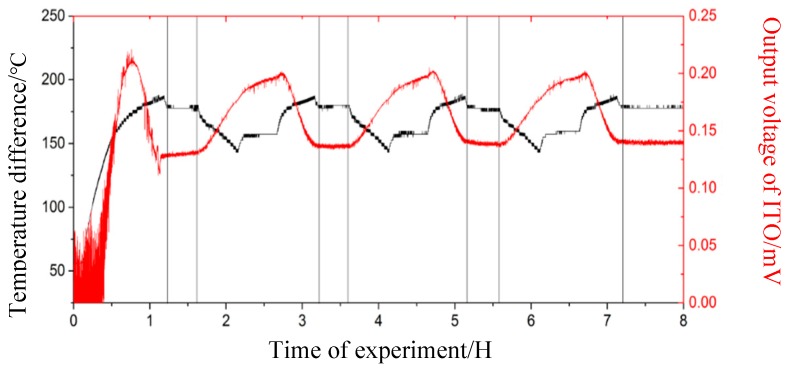
Relationship between the temperature difference and output voltage.

**Figure 12:**
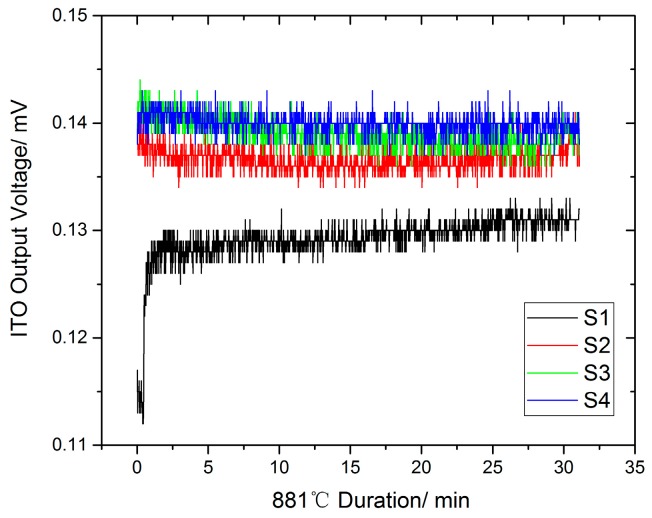
Heat cycle results.

During these experiments, the thermocouple output voltage value is recorded. The temperature difference between the working end and cold end is calculated based on the K-type thermocouple on both sides, as shown in Figure 10. The relationship between the temperature difference and the output thermoelectric voltage is obtained, as shown in Figure 11. Since the thermal inertia of turbine blade and the heat transfer processes on turbine blade, the temperature difference measured by standard thermocouples would not accord with the temperature difference load on turbine blade during the heating and cooling processes, but it would tend to be uniform during temperature holding process. For example, the output voltage of ITO thin film thermocouples will decrease at about 1 hour, and it is mainly caused by cold junction temperature on turbine blade increasing when the furnace holding at 1000 °C. To obtain more precisely thermoelectric properties of thin film thermocouples on turbine blade, we kept the furnace at 1000 °C for 30 min and choice the stable section as calibration results, as shown in Figure 12. It can ensure that the temperature difference load on ITO thin film thermocouple was precisely measured. With time going, the output voltage tends to be a constant value (0.14 mV), and it represents the reliable thermoelectric voltage of ITO thin film thermocouple under the temperature difference at 175 °C.

Combining the output voltage values of the K-type thermocouple and ITO thin-film thermocouple, the Seebeck coefficient of the ITO thin-film thermocouple can be obtained as follows:

$$\mathrm{Seebeck}\;\mathrm{coefficient}=\frac{\mathrm{Temperature}\;\mathrm{difference}}{\mathrm{Output}\;\mathrm{Voltage}}$$

Therefore, it is basically stable at 74.8 μV/°C when the temperature difference hold at 175 °C. Thus, the thermal insulation layer can establish an effective temperature balance and a feasible temperature calibration.

Figure 12 compares the thermal equilibrium state of the four temperature cycles. The second, third, and fourth cycles show that the average thermoelectric potential of the two consecutive heat preservation cycles is 0.14 mV at a high temperature of 881 °C. The output thermoelectric potential of the first cycle is slightly lower than that of the subsequent cycles, and tends to increase gradually. Thus, the variable cycle static test can determine that the stable output time of the ITO thin-film thermocouple signal on the turbine blade is 8 h.

## 5. Conclusions

In this study, the lead connection method between the lead wire and the thermocouple on a turbine blade is explored. The relationships between the wire bonding strength and influencing factors are explored experimentally, and the relationship between the roughness of the insulation layer and the contact resistance is determined. Stable data were obtained for 1000 °C thermal cycling tests, which confirms that the proposed through-hole lead connection technology can satisfy the test requirements. Subsequent research will focus on improving the joining method or joining materials and increasing the maximum temperature to simulate the real working environment of turbine blades. In the future, we will further cooperate with the aero-engine industry, and this type of lead wire should be tested by engine experiments to verify the reliability under operational conditions.

## Figures and Tables

**Table 1:** Factors of the orthogonal experiment.

Diameter of Through-Hole, D (mm)	Surface Roughness, Ra (μm)	Drying Temperature, T (°C)
0.35	0.84	80
0.5	1.19	120
0.7	1.97	160
1.0	2.24	200

**Table 2:** Orthogonal experiments and tensile measurement results.

Group	Diameter of Through-Hole, D (mm)	Surface Roughness, Ra (μm)	Drying Temperature, T (°C)	Tensile Strength, Fs (N)
1	350	0.85	80	29.9
2	350	1.19	120	5.5
3	350	1.97	160	28.6
4	350	2.24	200	14.6
5	500	0.84	160	18.7
6	500	1.19	200	16.5
7	500	1.97	80	18.3
8	500	2.24	120	15.1
9	700	0.84	200	17.4
10	700	1.19	160	17
11	700	1.97	120	12.3
12	700	2.24	80	14.6
13	1000	0.84	120	14.2
14	1000	1.19	80	6.7
15	1000	1.97	200	4.9
16	1000	2.24	160	3.2

**Table 3:** Significance analysis of the influence of orthogonal experimental factors.

Factor	Sum of Squares	F	Significance
*D*	345.262	2.548	*
*Ra*	196.007	1.447	*
*T*	86.347	0.637	-
*Error*	135.485		

**Table 4:** Resistance measurement results.

Group	Roughness, *Ra* (μm)	First Resistance Value (Ω)	Second Resistance Value (Ω)	Third Resistance Value (Ω)
R1	2.03	873.6	872.7	873.3
R2	1.25	619.5	619.7	619.5
R3	0.84	352.4	352.5	350.2
R4	0.46	98.95	99.16	98.85
